# On the incongruity between developmental plasticity and methodological rigidity

**DOI:** 10.3389/fnbeh.2012.00093

**Published:** 2013-01-17

**Authors:** Simone Macrì

**Affiliations:** Department of Cell Biology and Neuroscience, Section of Behavioural Neuroscience, Istituto Superiore di SanitàRoma, Italy

## Living organisms adjust their phenotypes according to environmental influences

One thousand nine hundred thirty six: this is the number of citations retrieved in “pubmed” using the search terms: “developmental plasticity rodent.” The “results by year” graphical trend, automatically plotted, indicates that this number has been linearly increasing over the last 30 years. Therefore, developmental plasticity, the range of different phenotypes potentially branching from an identical genotype (West-Eberard, 2003), is not a novel concept (Weininger et al., 1954). The notion that environmental factors modulate individual maturation, specifically during highly plastic developmental stages, is also not novel. Disciplines like psychology and evolutionary ecology devised theoretical and practical tools to understand the link between experiential factors and phenotypic adjustments. Whilst Freud proposed that adult neuroses build upon infantile experiences (Freud, 1918), contemporary authors demonstrated that several psychiatric disorders often root in early childhood, when abuse and/or neglect may increase individual vulnerability to depression (Heim and Nemeroff, 1999, 2001). These studies attempted to define the potentially disruptive nature of severe developmental stress. Complementary to them, other studies demonstrated that stress during development may represent a constructive force: specifically, moderate precocious environmental challenges have been proposed to favor resilience (Lyons and Macrì, 2011), i.e., program the organism to handle repeated stressors in a more efficient way. Studies conducted in rodents (Macrì et al., 2011), birds (Henriksen et al., 2011), primates (Parker et al., 2006; Parker and Maestripieri, 2011), and humans (DiCorcia and Tronick, 2011; Flinn et al., 2011; Seery, 2011), demonstrate that precocious exposure to mild stress (being briefly separated from dams during lactation, exposed to low doses of stress hormones, or reared to mothers requested to seek for food instead of being allowed unlimited foraging) promotes resilience.

At the same time that behavioral neuroscientists started disclosing the inextricable link between developing organisms and their environments, ethologists and evolutionary ecologists attempted to understand the functional meaning of these processes. Bateson and colleagues discussed the representative example of the freshwater crustacean *Daphnia* (Bateson et al., 2004). Offspring of this species develop a protective “helmet,” reducing the odds of being predated, if their mothers were exposed to a predator odor. Yet, the energetic costs associated with helmet patterning reduce individual competitive success in a predator-free environment. Thus, the success of each phenotype is dictated by the presence or absence of predators and, ultimately, by the correspondence (match) between neonatal forecasting and adult life conditions. A directional phenotypic adjustment in conformity with developmental cues has also been observed in rodents (Sachser, 1993; Sachser et al., 1994; Liu et al., 1997) and humans (Hales and Barker, 2001; Wells, 2007, 2011). The concept of resilience should be integrated within this theoretical framework, whereby precocious challenges may forecast an adult environment characterized by the presence of multiple stressors, to which the individual phenotype is accordingly tuned. Maladaptive or pathological outcomes may occur under several circumstances, among which the following two are of particular interest: (1) external challenges are too elevated to permit adaptive processes thereby exceeding individual adaptive capacities (Sultan, 2003); (2) developmental experiences do not provide a reliable indication of the challenges to be encountered later in life (phenotypic mismatch).

Along with the observation that experiential factors adjust individual development, so also the fundamental underlying mechanisms started being detailed. To investigate these mechanisms, laboratory rodents have often constituted the methodology of choice. For example, several studies demonstrated that being reared to a careful rat mother favored adult resilience through a non-genomic mother-offspring transfer mechanism (Francis et al., 1999). Specifically, increased adult resilience was shown to depend on maternally mediated epigenetic regulations at the level of DNA methylation (Weaver et al., 2004). Ultimately, laboratory animals constitute the cornerstone against which developmental plasticity is demonstrated and dissected.

I therefore find it quite ironic that such plasticity tends to be contrasted when it comes to using laboratory rodents as experimental subjects. Such contrast becomes particularly evident when current housing and breeding standards are considered. Thus, either in the case of conventional or enriched housing there exists a strong strive to equate living conditions across different facilities. Such strive is theoretically justified by the need to minimize and equalize environmental sources of variation to isolate the biological factors contributing to the individual phenotype, and to obtain reproducible results across different laboratories. I believe that these considerations entail several research questions: (1) does a unique laboratory standard produce identical individuals? (2) To what extent do laboratory rodents suit their environment? (3) If standardization were inefficient, what would the alternative be?

## Would a unique laboratory standard guarantee full reproducibility of experimental findings?

Under the assumption that different environments beget different phenotypes, it may be tenable to propose that a unique standard housing/breeding system should produce similar results. Before attempting to design a comprehensive digest listing how a rodent should be kept and tested (a hard duty), and select the committee devoted to this task (an even harder one), experimental support to the assumption that identical environments beget identical data should be obtained. This would require a set of identical experiments to be performed in independent facilities. Crabbe and colleagues performed such nobody-would-dare-to-do-experiment (Crabbe et al., 1999). The authors attempted to decompose the genetic and environmental influences on behavior through performing several tests in eight different mouse strains brought, kept, reared, and tested under the same conditions in three laboratories. Notwithstanding a spectacular level of across-lab standardization, the authors observed that mouse behavior was influenced by the test site, and concluded that “experiments characterizing mutants may yield results that are idiosyncratic to a particular laboratory.” The fact that this study involved the use of mutant mice is even more daunting as mutant mice derive from strains that have been mated with siblings for so many generations to become virtual genetic copies (for a discussion see Sapolsky, 2006). These results have also been replicated in an independent study (Wolfer et al., 2004).

Ultimately, identical subjects kept under “allegedly” identical environmental conditions may behave differently.

## To what extent do laboratory rodents suit their environment? can we imagine rearing conditions capable of promoting resilience?

Whilst evaluating whether behavioral data are reproducible across laboratories is attainable, determining whether laboratory rodents suit their environment is much more complex. Specifically, it is necessary to devise strategies aimed at evaluating whether rodents are adapted to their living conditions. Reproductive fitness may not constitute a biologically meaningful parameter in captivity, as hardly ever are laboratory rodents faced with contextual features capable of altering their life history strategies (the trade-offs between reproductive efforts and other fitness-relevant activities, like foraging and morphological growth, Del Giudice et al., 2011). Alternatively, we may address whether laboratory rodents exhibit behavioral abnormalities (reflecting brain dysfunctions) that are generally not displayed in natural conditions. Apparently functionless repeated behaviors (stereotypies) may constitute an informative parameter: up to 98% of ICR (Wuerbel et al., 1996) and 80% of C57BL/6 mice perform them under standard housing conditions (Garner, 2005). An elevated rate of abnormal spontaneous behavior has been proposed to constitute an index of poor welfare (Laviola et al., 1994; Gross et al., 2011). Additionally, to evaluate whether laboratory rodents are “normal,” it may be worth looking at the statistical distribution of the data collected upon them. Several studies show that experimental data are extremely variable and may greatly diverge from a “normal” distribution (Macrì et al., 2007). This has been proposed to stem from maladaptive adjustments to the laboratory environment (Garner, 2005) which, in turn, may relate to the fact that the neonatal living conditions do not constitute good predictors of the challenges to be encountered in adulthood (Wuerbel, 2001). Specifically, neonate laboratory rodents are exposed to extremely quiet, stable and safe conditions (the maximal source of stress being cage cleaning once/twice a week), including effortless *ad libitum* feeding conditions (unlikely to impose the foraging demands regularly met by a rodent dam in the wild). These conditions may not be good predictors of the continuous challenges to which rodents are often exposed in adulthood (e.g., injections, modified housing and re-grouping, food restrictions, etc.). In other words, laboratory rodents are not prepared to cope with the challenges imposed by laboratory routines. Thus, moderately challenging rearing conditions, aimed at promoting resilience, might prepare experimental subjects to efficiently handle the stressors associated with laboratory procedures, ultimately increasing experimental validity (reproducibility) and normalizing the statistical distribution of experimental data. To test these predictions, we exposed neonate mice to a supplementation of corticosterone (mimicking neonatal stress) and evaluated, in adulthood, the inter-individual variation and frequency distribution of data obtained in these individuals compared to standard laboratory controls. As predicted, adult mice exposed to challenging neonatal conditions exhibited reduced inter-individual variation across the following variables: anxiety-related behavior, pain perception, corticosterone response to restraint stress, and immune response to bacterial infection (Macrì et al., 2007). Thus, matching the stressful nature of the neonatal environment with actual adult test conditions may benefit the quality of laboratory data; by the same token, I believe that devising stress-free testing strategies (e.g., home-cage automated tasks: Galsworthy et al., 2005; Branchi et al., 2010; Voikar et al., 2010; Zoratto et al., 2012a,b) may benefit the quality of experimental data without requiring perinatal challenges to be administered to laboratory rodents.

## Can experimental requirements meet animal needs?

I previously discussed experimental studies in which current laboratory standards failed to yield reproducible results (Crabbe et al., 1999; Wolfer et al., 2004); I also proposed that breeding strategies aimed at “preparing” developing rodents to their future experimental habitat, may increase the reproducibility of experimental findings. Yet, this proposition is still clearly incomplete as it implies the use of animals kept and tested under univocal conditions.

Animal models generally attempt to test experimental hypotheses that pertain to a wide population, likely composed of variable individuals derived from different environments: a population in which developmental plasticity regularly occurs. Proposing a univocal standard would, by definition, neglect such plasticity. In analogy with phases 2-3-4 of clinical testing (in which the treatment is given to incrementally larger groups of people), I believe that pre-clinical experimental hypotheses should be tested in heterogeneous populations rather than in subsets of genetically and environmentally identical individuals. Therefore, future strategies shall include individual diversity in the experimental design across housing conditions, genetic predispositions and test paradigms. Theoretically, the potential influences of a given gene on a certain phenotype should be tested in mice derived from different strains rather than in a single background. Likewise, the effects of experimental variables should be tested in mice housed in systematically variable environmental conditions. This effort should be statistically supported by the adoption of random block experimental designs, allowing the analysis of the independent contribution (percentage of explained variance) exerted by the different factors involved. Several studies started investigating this possibility. Richter and collaborators demonstrated that data obtained in heterogeneous experimental populations, characterized by individuals derived from different stocks and breeding systems, yield more consistent results than studies involving the use of homogeneous groups (Richter et al., 2009, 2010, 2011).

Ultimately, I propose that future experimental approaches may implement the concepts discussed herein. Specifically, I foresee large-scale endeavors in which data are collected in systematically variable experimental populations (hetherogeneization); at the same time, experimental subjects constituting each statistical unit should be adapted to their specific test conditions (phenotypic match). Since an entirely stress-free laboratory environment cannot be applied on a large scale, the possibility to prepare rats and mice to multiple challenges should become a needed objective. It is thus necessary to devise diverse rearing systems capable of promoting laboratory-specific resilience. As researchers we are aware that, in order to dissect adaptive processes, we regularly expose experimental subjects to external sources of stressors. By the same token, we should acknowledge that analogous adaptive processes take place also when we are not specifically observing them, i.e., throughout the entire course of development. Rather than being neglected, individual plasticity should be incorporated in rearing and testing systems through the provision of consistent developmental information, matching precocious and adult environment.
